# Bioactive Collagen Peptides in Veterinary and Biomedical Science—Part I: Molecular Identity, Gastrointestinal Bioavailability, and Receptor-Mediated Signaling, with Relevance to the Bile Acid Axis

**DOI:** 10.3390/vetsci13080726

**Published:** 2026-07-23

**Authors:** Krisztián Németh, Marianna Kis, Borbála Mózes, Boglárka Mária Schilling-Tóth, Gergely Jócsák, István Tóth, Dávid Sándor Kiss, Katalin Lányi, Szilveszter Csorba, Tibor Bartha

**Affiliations:** 1 Department of Physiology and Biochemistry, University of Veterinary Medicine Budapest, István u. 2, H-1078 Budapest, Hungary; nemeth.krisztian@univet.hu (K.N.); jocsak.gergely@univet.hu (G.J.); toth.istvan@univet.hu (I.T.); kiss.david@univet.hu (D.S.K.); bartha.tibor@univet.hu (T.B.); 2 Csepel Central Veterinary Clinic, Táncsics Mihály u. 33, H-1212 Budapest, Hungary; hadakutjan@gmail.com; 3 Department of Internal Medicine, University of Veterinary Medicine Budapest, István u. 2, H-1078 Budapest, Hungary; mozes.borbala@univet.hu; 4 National Laboratory of Infectious Animal Diseases, Antimicrobial Resistance, Veterinary Public Health and Food Chain Safety, University of Veterinary Medicine Budapest, István u. 2, H-1078 Budapest, Hungary; 5 Institute of Food Chain Science, Food Chain Laboratory Center, University of Veterinary Medicine Budapest, Marek J. u. 2, H-1078 Budapest, Hungary; lanyi.katalin@univet.hu; 6 Institute of Food Chain Science, Department of Digital Food Science, University of Veterinary Medicine Budapest, István u. 2, H-1078 Budapest, Hungary; csorba.szilveszter@univet.hu

**Keywords:** bioactive collagen peptides, collagen hydrolysate, PEPT1/PEPT2, integrin signaling, Pro-Hyp, Nrf2, bile acid axis, FXR/TGR5, canine osteoarthritis, veterinary nutrition

## Abstract

Collagen, long regarded as the inert structural scaffold of the extracellular matrix, is now recognised as a source of bioactive signaling molecules: enzymatic hydrolysis releases low-molecular-weight bioactive collagen peptides that act systemically. This narrative review synthesises in vitro, animal-model, and veterinary clinical evidence with explicit differentiation of evidence levels; literature was identified through structured searches of PubMed, Web of Science, and Google Scholar, covering peer-reviewed, English-language reports published between 2000 and January 2026, without a formal systematic protocol. Across rodent, porcine, and human pharmacokinetic studies, orally administered collagen hydrolysate is efficiently absorbed, with a fraction reaching the circulation as intact prolyl-hydroxyproline and hydroxyprolyl-glycine through the conserved PEPT1/PEPT2 transporters; reported bioavailability varies with source, dose, and method, so no single value generalises across species. Native collagen and larger collagen fragments engage structure-dependent receptors in vitro (α2β1 integrins, DDR1/DDR2, GPVI, LAIR-1/2) that require triple-helical or Gly-Pro-Hyp presentation, whereas it is not established that the di- and tripeptides that reach the circulation after oral dosing engage them; their systemic actions are partly attributable to intracellular routes, including the Keap1–Nrf2 axis, HDAC/HAT modulation, and glycine-dependent glutathione synthesis. The gut–collagen peptide axis, a model derived from rodent and cell-culture data, links microbial bile acid remodeling and FXR/TGR5 signaling to systemic effects. Current evidence supports validated use in canine and equine osteoarthritis; species-specific bioavailability studies in dogs and cats remain the priority.

## Simple Summary

Collagen is the main structural protein of skin, bone, tendon, and cartilage, and it was long viewed only as a building material. When collagen is broken into small fragments—during digestion or in collagen supplements—some fragments are absorbed into the blood and act as signals that influence the body well beyond the tissues they came from. This review, the first of two parts, examines what these small collagen fragments are, how efficiently animals absorb them, and which cell-surface sensors and internal pathways they switch on. The evidence comes from laboratory experiments, animal studies, and clinical trials, and we are careful throughout to separate firm clinical findings from early laboratory observations. The strongest current evidence supports specific collagen fragments for relieving joint disease in dogs and horses. We also describe a newly recognised link between collagen fragments, gut bacteria, and bile that may explain effects on metabolism and the liver. The main message for practice is that collagen supplements are biologically active compounds whose effects differ between species, so the dose and the expected benefit should be judged species by species rather than assumed from one animal to another.

## 1. Introduction

### 1.1. Collagen Beyond Structure: Degradation Products as Signaling Peptides

Collagen was long treated as the inert, load-bearing scaffold of the metazoan extracellular matrix. Work over the past two decades has widened this view rather than overturned it: the enzymatic degradation products of this structural protein—specifically collagen hydrolysate and bioactive collagen peptides (BCPs)—also carry signaling activity. In experimental systems, these low-molecular-weight (LMW) sequences modify physiological, biochemical, and epigenetic readouts in mammalian, avian, and piscine cells and tissues. The systemic impact of collagen peptides extends beyond articular cartilage and dermal matrix remodeling [1]. Preclinical work—rodent models and cultured cells—implicates these peptides in gut–brain signaling, in immune modulation at the intestinal epithelium, and in the epigenetic programming of senescent fibroblasts [2,3]. None of these observations has yet been reproduced in a veterinary clinical population.

Collagen is the most abundant structural protein in vertebrates, comprising approximately 25–33% of total body protein mass. At least 28 distinct collagen types have been catalogued in mammals, classified by the molecular arrangement of their polypeptide chains. Type I collagen predominates in bone, skin, tendon, and vascular structures; Type II is the dominant collagen of hyaline articular cartilage; and Type III is characteristic of reticular fibers and early wound healing matrices. The triple-helical quaternary architecture of native collagen—three α-chains coiled around a common axis and stabilized by intramolecular hydrogen bonds and post-translational hydroxylation—renders the intact protein highly resistant to most endogenous proteases. This structural inertness, while advantageous in load-bearing tissues, has historically obscured the molecule’s biological potential [4].

Subsequent research demonstrated that specific hydrolytic processing—both physiological (by matrix metalloproteinases and cysteine cathepsins during tissue remodeling) and industrial (via targeted enzymatic hydrolysis)—liberates cryptic bioactive sequences from within the native collagen matrix. These so-called matricryptins or matrikines are peptide fragments that, upon release, expose receptor-binding domains hidden within the intact tertiary structure and transition from purely structural roles to active molecular signaling functions [1,5].

### 1.2. Systemic Bioactivity: Scope of the Review

The transition from intact whole collagen to bioactive peptides is dictated by specific hydrolytic processes that yield fragments capable of surviving gastrointestinal transit, entering the systemic circulation, and binding to highly specific cellular receptors [4]. This requires a rigorous, multidisciplinary evaluation of how exogenously administered collagen peptides interact with host physiology. Synthesizing recent veterinary clinical evidence and human biomedical models clarifies how specific peptide sequences can be applied—from the mitigation of degenerative joint disease in canines and equines [6], to the correction of metabolic dysfunction in obesity models, and the potential reversal of fibrotic cascades in hepatic tissue [7,8,9].

The commercial production of collagen hydrolysates (CH) is a heterogeneous process, yielding complex mixtures of oligopeptides and free amino acids with a low mean molecular weight—typically 2–6 kDa—which ensures high digestibility, rapid absorption, and a very low risk of inducing feed-induced allergic responses [10]. Raw materials and processing methods determine CH characteristics: the amino acid composition of collagens is species-specific, and the degree of collagen denaturation and hydrolysis—determined by the proteolytic enzymes selected, incubation time, and processing temperature—directly influences the resulting oligopeptide profile and bioavailability [4].

A critical distinction must be maintained between collagen hydrolysate (CH), which is fully soluble and contains diverse short-chain peptides predominantly below 3000 Da, and undenatured (native) Type II collagen (UC-II), which retains its intact triple-helical quaternary structure. These two product classes exert bioactivity through entirely different mechanisms: CH primarily acts through direct receptor-mediated signaling at the cellular level following systemic absorption, while UC-II operates via oral tolerization of the immune system at the intestinal mucosal level through gut-associated lymphoid tissue (GALT) [11,12,13].

Three terms are used throughout this review with fixed meanings. Collagen hydrolysate (CH) denotes the ingested product: an enzymatically hydrolyzed, fully soluble mixture of oligopeptides and free amino acids. Bioactive collagen peptides (BCPs) denote the peptide sequences within or released from that mixture for which biological activity has been measured, principally Pro-Hyp and Hyp-Gly. Undenatured type II collagen (UC-II) denotes the non-hydrolyzed, triple-helical preparation, which is not a hydrolysate and does not act through systemic absorption. The three are compared in Table 1. Commercial usage conflates these terms; this review does not.

### 1.3. Aims and Scope of This Review; Stratification of Evidence Levels

This comprehensive narrative review evaluates the molecular identity, receptor-mediated mechanisms, and the translational applications of bioactive collagen peptides, with specific emphasis on veterinary relevance. Throughout this manuscript, evidence is explicitly stratified by its methodological origin: (1) in vitro cellular and biochemical assays, which establish mechanistic plausibility but cannot be directly extrapolated to clinical outcomes; (2) laboratory animal models (rodent, lagomorph), which provide in vivo proof-of-concept but require translational validation; and (3) veterinary clinical trials in target species (canine, equine, feline), which represent the highest level of directly applicable evidence for the practicing veterinarian.

This distinction is not simply academic. A considerable portion of the claims surrounding collagen peptide supplementation is based on in vitro data obtained at supraphysiological concentrations or under highly controlled cell culture conditions that may not reflect the complex milieu of the living organism. A critical synthesis stratifying the evidence hierarchy is therefore essential for evidence-based clinical application.

Literature was identified by structured searches of PubMed, Web of Science, and Google Scholar. Search terms combined (“collagen peptide” OR “collagen hydrolysate” OR “bioactive collagen peptide” OR gelatin) with (bioavailability OR PEPT1 OR integrin OR “discoidin domain receptor” OR Nrf2 OR “bile acid”) and with species terms (dog, cat, horse, pig, poultry). Records published between 2000 and January 2026 were screened, and only peer-reviewed, English-language reports were retained. Reference lists of retrieved reviews were hand-searched. No formal systematic protocol, no risk-of-bias instrument, and no duplicate screening were applied; selection bias therefore cannot be excluded, and the coverage should be read as broad but not exhaustive. Each substantive claim below is labeled by evidence type—in vitro, laboratory animal model, veterinary clinical trial, or human clinical trial—and this labeling is applied consistently in Section 2 and Section 3, and 4, including where mechanistic and clinical material appear in the same paragraph. Translational and clinical applications are treated in Part II of this series.

## 2. Molecular Identity and Bioavailability

### 2.1. Structural Biology and Enzymatic Hydrolysis Parameters

Type I collagen, the most abundant protein in the mammalian body, is characterized by its complex hierarchical structure. The primary sequence is dominated by a repeating Gly-X-Y motif, where X and Y are frequently occupied by proline (Pro) and hydroxyproline (Hyp), respectively. This specific amino acid profile—comprising roughly 33% glycine and 22% Pro and Hyp—spontaneously coils into a left-handed poly-L-proline II type helix. Three such α-chains, each containing approximately 1050 amino acid residues, intertwine to form the rigid, right-handed triple superhelix that constitutes the tertiary and quaternary structures of native tropocollagen. The inherent stability of this molecule, fortified by extensive intramolecular hydrogen bonding between glycine residues and post-translational enzymatic cross-linking by lysyl oxidase, renders native collagen highly resistant to standard proteolytic digestion in the mammalian gastrointestinal tract [4].

To overcome this proteolytic resistance and activate the signaling pathways of the protein, industrial and pharmaceutical processing employs targeted extraction and hydrolysis [4,16]. Extraction methods predominantly involve chemical (acid or alkaline pre-treatments) or physical (hydrothermal) approaches to denature the quaternary structure into soluble gelatin. Following denaturation, specific proteases—such as alcalase, trypsin, pepsin, or the multi-enzyme preparation Protamex—are introduced to cleave the massive 300 kDa tropocollagen into low-molecular-weight collagen peptides, typically targeting a range of 2 to 6 kDa [4,17].

In vitro manufacturing optimization models demonstrate that altering the enzymatic incubation time and temperature directly dictates the bioactivity of the resulting hydrolysate. For instance, treatment of marine collagen with trypsin for exactly three hours yields peptides with maximum angiotensin-I-converting enzyme (ACE) inhibitory activity, while prolonged hydrolysis alters the peptide sequence and diminishes receptor binding affinity [4]. A critical practical consideration is that CH products with different mean molecular weights—whether bovine high-molecular-weight (HMW, ~5000 Da) or low-molecular-weight (LMW, ~2000 Da)—yield highly comparable peptide and amino acid concentrations in blood following oral ingestion, suggesting that di- and tripeptides are further released during gastrointestinal digestion and absorption regardless of the initial product molecular weight [16,18].

Batch-to-batch variability is a practical constraint that is rarely quantified. Degree of hydrolysis, enzyme lot activity, raw-material age, and post-hydrolysis fractionation each shift the peptide molecular-weight distribution, and manufacturers seldom publish lot-level peptide profiles. Two products carrying the same label claim can therefore differ in the abundance of the sequences that carry the measured bioactivity. Because clinical trials almost never report lot-level characterization of the preparation used, differences in outcome between trials cannot be separated from differences between batches [4,10,18].

A comparison of industrial hydrolysis parameters and the resulting bioactive peptide profiles across major collagen sources is provided in Table 2. The values are approximate ranges compiled from the sources cited in each column, not standardized product specifications; commercial products vary widely around them, and no regulatory standard harmonizes them.

### 2.2. Species-Specific Origins and Amino Acid Profiles

The biological activity of the resulting peptides is directly dependent on the species of origin. Traditional mammalian sources, primarily bovine and porcine hides, provide a matrix of Type I and Type III collagens characterized by high thermal stability and dense hydroxyproline cross-linking. However, zoonotic concerns (such as bovine spongiform encephalopathy, BSE) and religious dietary restrictions have driven the rapid exploration of alternative raw materials [20].

Marine sources—including chum salmon (*Oncorhynchus keta*), tilapia skin, barred mackerel, jellyfish (*Rhopilema esculentum*), and eel bone (*Anguilla japonica*)—represent highly bioavailable and safe alternatives that substantially reduce the risk of mammalian pathogen transmission—including bovine spongiform encephalopathy (BSE), foot-and-mouth disease (FMD), and transmissible spongiform encephalopathies (TSEs)—compared with bovine or porcine sources; this lower mammalian-pathogen risk does not amount to freedom from microbiological or environmental contamination, which remains dependent on harvest and processing conditions compared with bovine or porcine sources. They are not, however, free of microbiological or chemical risk: marine raw materials can carry spoilage organisms, marine biotoxins, and accumulated heavy metals, and the residual risk depends on harvest site, storage, and processing conditions [20,22]. Proteomic analysis reveals that piscine-derived collagen proteins generally exhibit lower absolute levels of Pro and Hyp compared to mammalian collagens, resulting in a characteristically diminished thermal denaturation temperature (typically 15–20 °C lower than bovine equivalents). The lower denaturation temperature has a practical consequence: gelatin can be extracted from fish skin at a lower temperature than from bovine hide, which reduces the energy required. The same property limits the thermal stability of the resulting product [20].

Marine collagen peptides frequently possess unique hydrophobic amino acid sequences that exhibit superior antioxidant capacities and specialized receptor affinities compared to their mammalian counterparts. Collagen peptides from jellyfish (*R. esculentum*) demonstrated potent antioxidant activity—including lipid oxidation inhibition, superoxide anion-scavenging, hydroxyl radical-scavenging, and copper-chelating activities—in concentration-dependent assays, alongside melanogenesis-inhibitory activity via suppression of intracellular tyrosinase expression [21]. Poultry by-products, including chicken feet and sternum cartilage, provide abundant sources of Type II collagen, critical for specialized applications in articular joint immunology [11]. Advanced liquid chromatography-tandem mass spectrometry (LC-MS/MS) techniques are increasingly employed to distinguish species-specific marker peptides, supporting product authentication in the companion animal patient.

**Figure 1:**
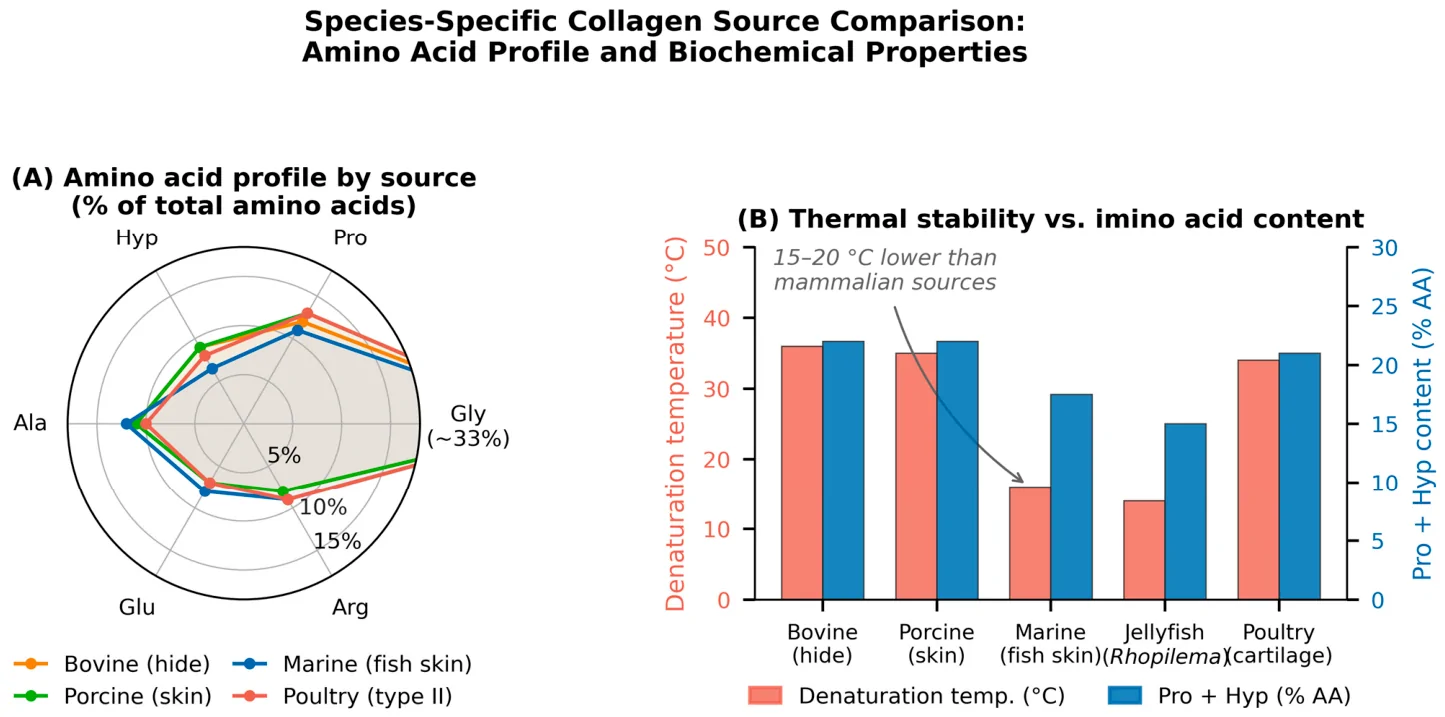
Species-Specific Collagen Source Comparison: Amino Acid Profile and Biochemical Properties. (**A**) Radar chart comparing the relative amino acid (AA) composition of collagen from four major commercial sources (bovine, porcine, marine, and poultry); values expressed as percentage of total AA residues. (**B**) Paired bar chart comparing denaturation temperature (°C) and Pro + Hyp content (%) across these sources together with jellyfish (*Rhopilema*) collagen; marine and jellyfish collagens display 15–20 °C lower denaturation temperatures than mammalian equivalents, facilitating lower-temperature industrial processing. Abbreviations: AA, amino acid; Pro, proline; Hyp, hydroxyproline; °C, degrees Celsius.

The relationship between dietary fiber, bile acid remodeling, and FXR/TGR5 signaling—shown in recent canine psyllium data—is examined in Part II (Section 2.2); the relevant point here is that collagen hydrolysate is typically co-administered with other bile acid-active dietary components, which complicates attribution of systemic effects. The amino acid profiles and thermal properties of major collagen sources are depicted in Figure 1.

### 2.3. Gastrointestinal Absorption and Pharmacokinetics

The bioavailability of orally administered collagen peptides modifies the traditional model of dietary protein absorption, which holds that proteins are reduced to free amino acids prior to intestinal absorption. Pharmacokinetic studies using murine and porcine vascularly perfused intestinal and hepatic preparations report relative and absolute bioavailabilities of 74.12% and 85.97%, respectively, in rats [23]; these describe total absorption of the collagen-derived material, most of which enters as free amino acids. In the same rat study, the plasma amino acid profile indicated that 41.91% of the digested gelatin was absorbed as peptide rather than as free amino acids [23]. In human volunteers, the peptide-bound fraction is lower and source-dependent: the integrated hydroxyproline AUC exceeded that of free hydroxyproline by 47% for fish and porcine, 43% for bovine low-molecular-weight, and 36% for bovine high-molecular-weight hydrolysate [18,19]. Because these values come from perfused-organ, single-source studies, they bound the intact-peptide fraction in those models rather than defining a general bioavailability for collagen peptides.

The observation that orally administered collagen hydrolysate is absorbed and accumulates in cartilage, together with the associated early clinical literature, was first drawn together two decades ago [24]; the four open-label and three double-blind human studies available at that time were of variable methodological quality, and the mechanistic account below rests on work published since.

In a human randomized, double-blind, crossover clinical trial assessing single-dose bioavailability of CH from multiple sources (porcine, bovine, and fish skin/hide), the ingestion of CH yielded rapid and highly significant elevations in circulating free and peptide-bound hydroxyproline [18]. The pharmacokinetics demonstrate a distinct peak in plasma dipeptides and tripeptides—primarily Pro-Hyp and Hyp-Gly—within 60–120 min post-ingestion, returning to baseline concentrations within 24 h. The higher concentration of total Hyp compared to free Hyp in serum confirms the systemic uptake of intact oligopeptides [25,26]. Independently of source (bovine, porcine, or fish) and initial molecular weight (2000–5000 Da), all CH formulations yielded comparable plasma concentrations of Pro-Hyp and other metabolites, demonstrating that pharmacokinetic outcome is primarily determined by gastrointestinal digestion rather than initial product specification [18].

The absolute exposures involved are small. Peak Pro-Hyp concentrations after a 10 g dose lie in the low μg/mL range, at least an order of magnitude below the concentrations at which most receptor-level effects have been demonstrated in cell culture (Section 4.3.1). Whether these circulating concentrations are sufficient to produce the cellular responses attributed to them is an open question and not a settled one.

**Figure 2:**
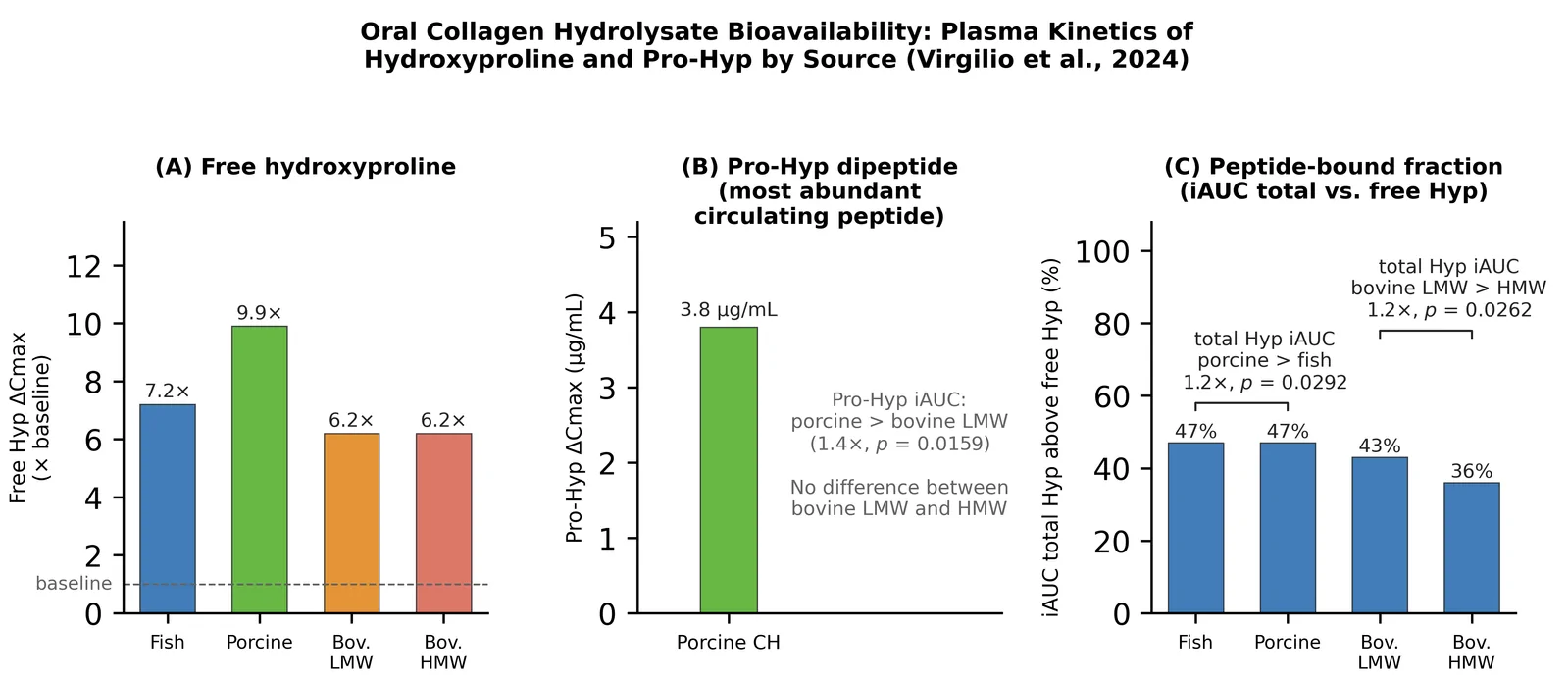
Oral Collagen Hydrolysate Bioavailability: Plasma Kinetics of Free Hydroxyproline and Key Bioactive Dipeptides by Source [18]. (**A**) Peak plasma free-hydroxyproline response after single-dose ingestion of 10 g collagen hydrolysate from four sources, expressed as the fold-increase in ΔCmax over baseline (7.2× fish, 9.9× porcine, 6.2× both bovine preparations). (**B**) Peak plasma Pro-Hyp concentration (ΔCmax, μg/mL); porcine collagen hydrolysate reached 3.8 μg/mL and produced a significantly higher integrated Pro-Hyp exposure than the bovine low-molecular-weight preparation (1.4-fold, *p* = 0.0159). (**C**) Integrated hydroxyproline exposure absorbed in peptide-bound form, expressed as the percentage by which total-hydroxyproline iAUC_0–360_ exceeded free-hydroxyproline iAUC (47% fish, 47% porcine, 43% bovine LMW, 36% bovine HMW); porcine exceeded fish (1.2-fold, *p* = 0.0292) and bovine LMW exceeded bovine HMW (1.2-fold, *p* = 0.0262). Data are presented as mean ± SD, n = 6. Abbreviations: CH, collagen hydrolysate; LMW, low molecular weight; HMW, high molecular weight; Hyp, hydroxyproline; Pro-Hyp, prolyl-hydroxyproline; ΔCmax, baseline-corrected maximum plasma concentration; iAUC, integrated area under the curve; SD, standard deviation.

An important mechanistic observation is that the initial peptide content of a CH product is not a direct indicator of expected blood concentrations of a given peptide after ingestion. Pro-Hyp appears at very high plasma concentrations (porcine CH ΔCmax ~3.8 μg/mL) despite being present at relatively low abundance in the pre-ingested product, whereas Gly-Pro—present at the highest abundance in the CH product prior to ingestion—resulted in circulating concentrations approximately 100-fold lower. This difference indicates that Pro-Hyp is primarily released from larger oligopeptides during gastrointestinal transit and mucosal absorption, rather than being pre-formed in the ingested supplement [18]. Following systemic absorption, oral administration of CH with radiolabeled Pro or Pro-Hyp in rodent models led to accumulation of radioactivity in cartilage, bone, and the synovium, directly demonstrating the tissue-specific targeting of circulating BCP derivatives [10]. The pharmacokinetic profiles are illustrated in Figure 2.

### 2.4. PEPT1/PEPT2 Transporter Mechanics and Plasma Stability

The transcellular absorption of intact oligopeptides from the intestinal lumen is primarily mediated by proton-dependent oligopeptide transporters, specifically PEPT1 (SLC15A1) and PEPT2 (SLC15A2). These electrogenic symporters are expressed abundantly on the brush border membrane of enterocytes and utilize an inwardly directed transmembrane electrochemical proton gradient—maintained by the Na+/H+ exchanger NHE3—to efficiently shuttle di- and tripeptides into the portal circulation [27,28]. PEPT1 and PEPT2 belong to the highly conserved proton-dependent oligopeptide transporter (POT/SLC15) family—members of the major facilitator superfamily characterized by a 12-transmembrane-domain architecture and conserved substrate-binding residues that confer di- and tripeptide specificity across mammalian orthologs [27,29,30]—providing a mechanistic basis for cross-species comparison of the absorption mechanism. Conservation of the transporter is not the same as equivalence of pharmacokinetics: gastric and intestinal pH, transit time, brush-border peptidase activity, meal composition, and feeding pattern all determine how much intact peptide reaches the portal blood, and these differ between dogs, cats, pigs, horses, and humans. Canine and porcine PEPT1 have been cloned and functionally characterized and retain the architecture and substrate specificity of the human orthologs; the conservation is qualitative, not a numeric sequence identity.

Conservation of the transporter supports cross-species comparison of the absorption route, not the direct transfer of human pharmacokinetic values to veterinary species. However, dogs present with meaningful differences in gastrointestinal physiology relative to humans—including faster small intestinal transit time, a higher and more variable intestinal luminal pH, and a distinct pattern of brush border enzyme activity distributed along proximo-distal gradients that differs from the human profile [31,32]—and specific pharmacokinetic studies characterizing BCP tissue distribution following oral dosing in dogs remain a recognized gap in the literature requiring targeted investigation.

**Figure 3:**
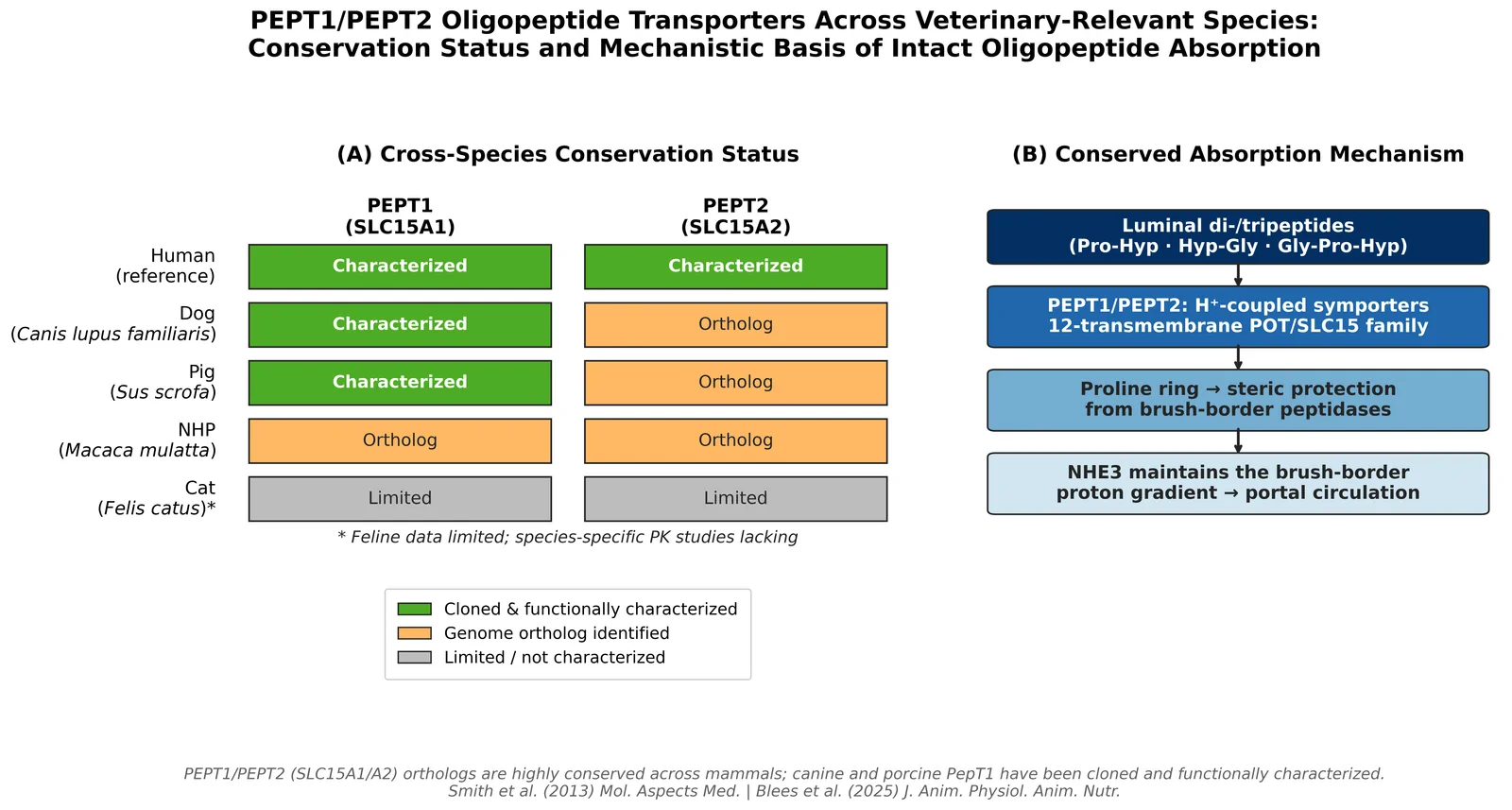
Conservation of PEPT1 and PEPT2 Oligopeptide Transporters Across Veterinary-Relevant Species. (**A**) Qualitative cross-species conservation status of PEPT1 (SLC15A1) and PEPT2 (SLC15A2): for each species, the transporter is classified as functionally characterized (cloned and functionally expressed), genome ortholog identified, or limited/not characterized. (**B**) Schematic of the conserved intestinal absorption mechanism: luminal di-/tripeptides are taken up by the H^+^-coupled symporters PEPT1/PEPT2 (12-transmembrane POT/SLC15 family); and the NHE3-maintained proton gradient drives transport into the portal circulation. * Feline data are limited; species-specific pharmacokinetic studies remain lacking (Smith et al., 2013; Blees et al., 2025) [10,27]. Abbreviations: PEPT1, peptide transporter 1; PEPT2, peptide transporter 2; SLC15A1/A2, solute carrier family 15, members 1 and 2; POT, proton-dependent oligopeptide transporter; NHE3, sodium–hydrogen exchanger 3; NHP, non-human primate.

Upon reaching the systemic circulation, Pro-Hyp and related peptide fragments demonstrate notable biological stability [25]. The unique cyclic pyrrolidine ring structure of proline and hydroxyproline provides significant steric hindrance to peptidase attack, conferring substantial resistance against cleavage by native blood plasma peptidases—a property not shared by peptides of non-collagenous origin. Consequently, these circulating di- and tripeptides achieve sufficient half-lives to be deposited into target tissues—including articular cartilage, the dermal ECM, and endothelial linings—where they transition from circulating nutritional metabolites to active molecular signaling agents at specific receptor sites. The interspecies conservation of the PEPT1/PEPT2 system is illustrated in Figure 3. In panel (B) of Figure 3, the box shading denotes sequential steps only and carries no categorical meaning.

## 3. Signal and Receptor-Mediated Roles

**Figure 4:**
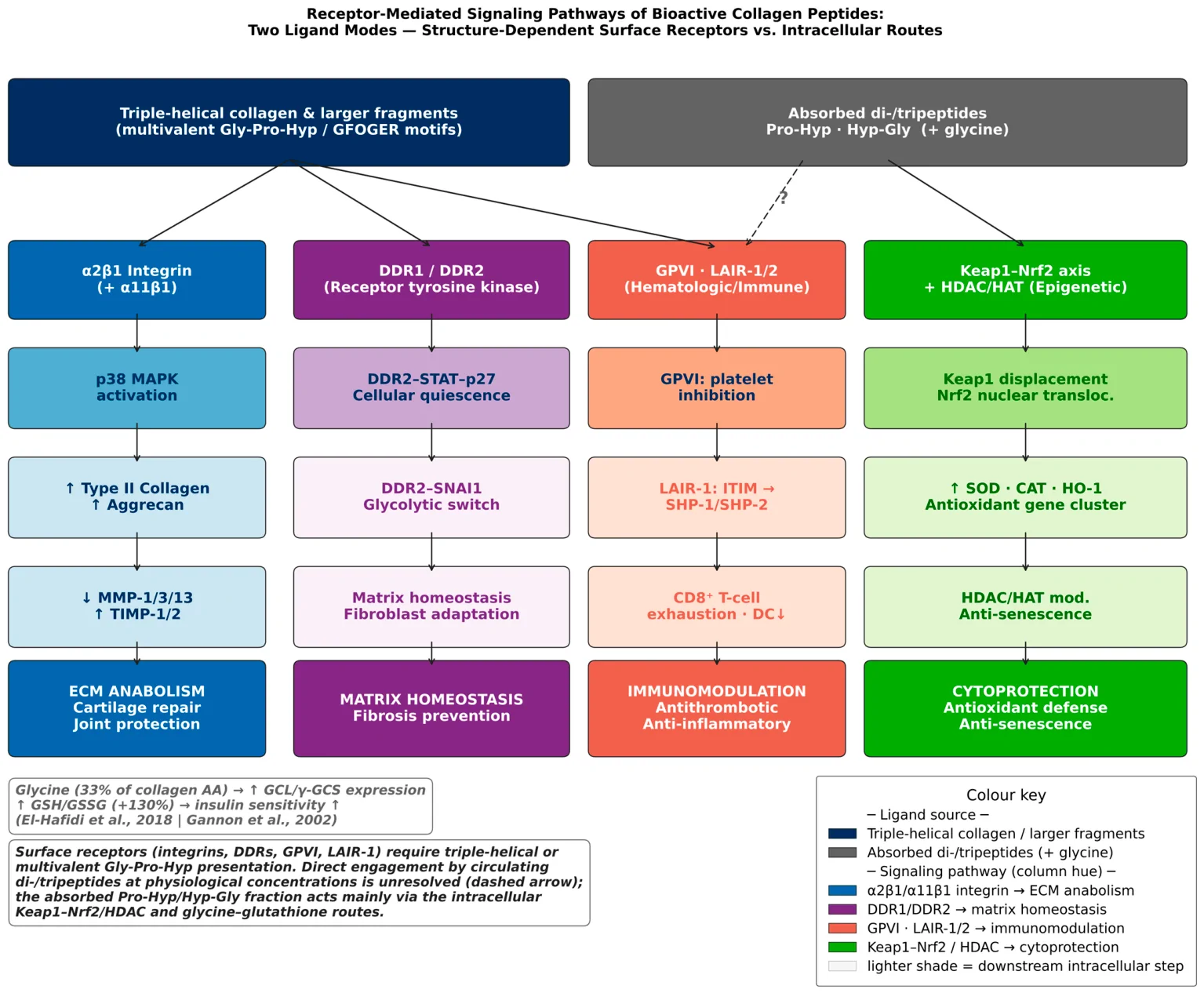
Receptor-Mediated Signaling Pathways of Bioactive Collagen Peptides: Two Ligand Modes—Structure-Dependent Surface Receptors versus Intracellular Routes. Collagen and collagen-derived peptides act through two distinct ligand modes. Triple-helical collagen and larger fragments engage the structure-dependent surface receptors (α2β1/α11β1 integrins, DDR1/DDR2, GPVI, LAIR-1), which require multivalent triple-helical or repetitive Gly-Pro-Hyp/GFOGER presentation; the absorbed di-/tripeptides (Pro-Hyp, Hyp-Gly) act predominantly through intracellular routes, principally the Keap1–Nrf2/HDAC axis. In the diagram, solid arrows carry two roles: those descending from the two ligand-source boxes to the receptor columns show which ligand mode engages which receptor class, and those running down each column show the order of the intracellular cascade, each step leading to the one below and ending in the functional outcome (bottom row). The single dashed arrow, marked with a question mark, denotes the one proposed but experimentally unconfirmed route: direct engagement of the GPVI and LAIR-1/2 surface receptors by absorbed di-/tripeptides at physiological plasma concentrations (El-Hafidi et al., 2018; Gannon et al., 2002) [33,34]. Abbreviations: BCP, bioactive collagen peptide; Pro-Hyp, prolyl-hydroxyproline; Hyp-Gly, hydroxyprolyl-glycine; Gly-Pro-Hyp, glycine-proline-hydroxyproline; DDR1/2, discoidin domain receptor 1/2; GPVI, glycoprotein VI; LAIR-1/2, leukocyte-associated immunoglobulin-like receptor 1/2; MAPK, mitogen-activated protein kinase; MMP, matrix metalloproteinase; TIMP, tissue inhibitor of metalloproteinases; SNAI1, snail family transcriptional repressor 1; STAT, signal transducer and activator of transcription; ITIM, immunoreceptor tyrosine-based inhibitory motif; SHP-1/2, Src homology 2-containing protein tyrosine phosphatase 1/2; Keap1, Kelch-like ECH-associated protein 1; Nrf2, nuclear factor erythroid 2-related factor 2; ARE, antioxidant response element; SOD, superoxide dismutase; CAT, catalase; HO-1, heme oxygenase-1; HDAC, histone deacetylase; HAT, histone acetyltransferase; ECM, extracellular matrix; GSH, glutathione; GSSG, oxidized glutathione; GCL, glutamate-cysteine ligase; γ-GCS, γ-glutamylcysteine synthetase.

The bioactivity of collagen peptides is not only a function of providing raw amino acid substrates for endogenous protein synthesis. Instead, the evidence strongly supports a complex, receptor-mediated signaling framework. Specific oligopeptides act as matricryptins—cryptic bioactive sites hidden within the native three-dimensional structure of the ECM that become exposed and biologically active only upon enzymatic hydrolysis or physiological degradation [1]. Four major receptor classes mediate this collagen sensing (Figure 4). A structural caveat applies throughout this section: integrins, DDRs, GPVI, and LAIR-1 recognize collagen through its triple-helical conformation and repetitive Gly-Pro-Hyp/GFOGER motifs, presented multivalently. This binding mode is satisfied by intact collagen, by matrix-embedded collagen, and by larger fragments, but not necessarily by the free di- and tripeptides (Pro-Hyp, Hyp-Gly) that make up the orally absorbed fraction. The mechanisms below therefore describe how cells sense collagen and collagen fragments; the extent to which circulating di-/tripeptides reproduce these interactions in vivo is addressed in Section 4.3.

Unless stated otherwise, the receptor-level findings in Section 3.1, Section 3.2 and Section 3.3 derive from in vitro systems using intact collagen, matrix-embedded collagen, or synthetic triple-helical peptides, not from oral administration of collagen hydrolysate.

In Figure 4, hue denotes the signaling pathway (blue, integrin; purple, DDR1/DDR2; red, GPVI and LAIR-1/2; green, Keap1–Nrf2/HDAC), and lighter shades within a column denote downstream intracellular steps. The four pathways initiate distinct but convergent anabolic, homeostatic, immunomodulatory, and cytoprotective cascades.

### 3.1. Integrin-Mediated Cellular Signaling

Collagen-derived peptides act as direct ligands for multiple cell-surface receptors, the most prominent being the integrin family. Integrins are heterodimeric transmembrane glycoproteins composed of non-covalently associated alpha and beta subunits; collagen peptides primarily target the β1 integrin subfamily, with the α2β1 and α11β1 heterodimers representing the principal receptors on mesenchymal cells, fibroblasts, and chondrocytes.

In vitro cellular assays demonstrate that engagement of the α2β1 integrin by triple-helical collagen and GFOGER-type motifs triggers outside-in signaling cascades [35]. This interaction induces the isoform-specific activation of p38 mitogen-activated protein kinase (MAPK) via a mechanism specifically involving the α2 cytoplasmic tail—distinct from the ERK1/2 pathway activated by fibronectin [35]. The activation of the p38 MAPK pathway directly upregulates endogenous type II collagen and aggrecan gene transcription in chondrocytes [35], and modulates the balance between ECM synthesis and degradation by suppressing the expression of matrix metalloproteinases (MMP-1, MMP-3, MMP-13) while simultaneously upregulating their endogenous inhibitors (TIMP-1, TIMP-2) [10,14,17]. This describes a feed-forward loop available to cells exposed to triple-helical collagen or to GFOGER-presenting fragments. It has not been shown that orally administered collagen hydrolysate reaches chondrocytes in a form capable of engaging α2β1, and the clinical improvement recorded in canine and equine osteoarthritis (Part II) is consistent with this route without demonstrating it.

### 3.2. Discoidin Domain Receptors (DDR1/DDR2) and Matrix Homeostasis

Parallel to integrin signaling, collagen peptides interface with discoidin domain receptors (DDR1 and DDR2). Unlike integrins, DDRs are unique receptor tyrosine kinases that specifically recognize collagenous matrices and function independently of the integrin pathways, providing a complementary but non-redundant signaling arm. DDR2 is the predominant isoform on chondrocytes and fibroblasts within the synovial joint and articular cartilage microenvironment.

Advanced molecular studies reveal that the DDR complexes modulate long-term cellular responses at the transcriptional level [1]. Specifically, the COL-I–DDR2–STAT–p27 signaling axis is responsible for maintaining cellular quiescence in fibroblasts—a protective state preventing pathological hyperproliferation in chronic inflammatory conditions—while the COL–DDR2–SNAI1 axis mediates metabolic shifts toward glycolysis in specific tissue microenvironments, including those found in activated synoviocytes during rheumatoid arthritis progression [1]. This receptor system ensures that fibroblasts and chondrocytes adapt their metabolic output and proliferative state to match the structural demands of the surrounding degraded matrix, positioning DDR signaling as a key homeostatic sensor within the ECM.

The DDR literature derives largely from tumor biology, fibrosis, and tissue-remodeling models in which collagen is present as an intact matrix. Its relevance to nutritional collagen supplementation is inferential: no study has shown that dietary collagen peptides alter DDR signaling in vivo.

### 3.3. Immune-Modulatory and Hematologic Receptors (GPVI and LAIR-1/LAIR-2)

Beyond structural tissue remodeling, circulating collagen peptides directly interface with the hematologic and immune systems [36]. Glycoprotein VI (GPVI), a type I transmembrane protein predominantly expressed on megakaryocytes and platelets, is a critical collagen receptor that mediates platelet activation, aggregation, and thrombus formation under normal physiological conditions. The GPVI ectodomain contains two Ig-like loops that bind specifically to collagen triple-helical sequences, particularly those containing the GPO (Gly-Pro-Hyp) motif [36].

In vitro hematologic models show that synthetic collagen-related peptides—crosslinked Gly-Pro-Hyp constructs mimicking the triple-helical GPO repeat—can occupy GPVI and modulate platelet reactivity, of potential atheroprotective and antithrombotic interest; these constructs are multivalent and triple-helical, and whether the monomeric di-/tripeptides absorbed from oral collagen hydrolysate reproduce this effect at physiological concentrations has not been established [36]. Platelet hyperreactivity in diabetic dogs and cats makes this receptor of interest in veterinary medicine, but no collagen hydrolysate study has addressed it.

Independently, collagen fragments engage leukocyte-associated immunoglobulin-like receptors (LAIR-1 and LAIR-2, a secreted decoy receptor), which function as potent inhibitory immune receptors on natural killer cells, dendritic cells, mast cells, and T lymphocytes. Collagen and collagen-derived fragments presenting repetitive Gly-Pro-Hyp motifs engage LAIR-1 on immune cell surfaces, inducing an immunosuppressive signal transmitted through intracellular immunoreceptor tyrosine-based inhibitory motifs (ITIMs) and the recruitment of SHP-1 and SHP-2 phosphatases. This inhibitory pathway promotes CD8+ T cell exhaustion, dampens hyperactive inflammatory cascades, and modulates dendritic cell maturation via the CAMK1-CREB signaling pathway [1].

Both GPVI and LAIR-1 have been engaged experimentally only by triple-helical, multivalent ligands. Neither has been shown to respond to the monomeric di- and tripeptides that dominate the absorbed fraction, and no nutraceutical study has measured GPVI or LAIR-1 occupancy after oral collagen hydrolysate. This section therefore describes receptor biology, not a demonstrated effect of supplementation.

### 3.4. Intracellular Effects: Keap1–Nrf2 and HDAC/HAT Modulation, and Glycine-Dependent Glutathione Synthesis

Recent studies identify specific peptide sequences as direct epigenetic modulators capable of reprogramming the transcriptional program of senescent and damaged cells [37]. Age-related and pathology-induced degradation of the ECM disrupts epigenetic homeostasis, driving cells toward a senescence-associated secretory phenotype (SASP) characterized by sustained expression of pro-inflammatory interleukins, MMPs, and chemokines.

Select bioactive sequences derived from marine sources modulate the Kelch-like ECH-associated protein 1 (Keap1), which under basal conditions retains nuclear factor erythroid 2-related factor 2 (Nrf2) in the cytoplasm and targets it for proteasomal degradation [38]. Marine collagen peptides activate the Keap1–Nrf2 axis, promoting Nrf2 nuclear translocation. Once in the nucleus, Nrf2 binds antioxidant response elements (AREs) and triggers the transcription of an extensive endogenous antioxidant defense network, including superoxide dismutase (SOD), catalase (CAT), glutamate-cysteine ligase (GCL), and heme oxygenase-1 (HO-1) [38,39].

Concurrently, specific collagen peptides regulate the activity of histone deacetylases (HDACs) and histone acetyltransferases (HATs), directly modulating chromatin accessibility and the transcriptional activity of pro-regenerative gene clusters [37]. In cortisol-treated senescent human dermal fibroblasts—a model of glucocorticoid-induced skin aging—AP collagen peptides rich in Gly-Pro-Hyp (GPH) tripeptides prevented the cortisol-mediated downregulation of type I procollagen production by blocking glucocorticoid receptor (GR)-mediated signaling and the downstream TGF-β inhibitory cascade [37,40].

The studies underpinning this section used purified glycine, defined synthetic peptides, or laboratory-fractionated marine peptides rather than commercial collagen hydrolysate. Whether a commercial product delivers the same sequences at the same concentrations has not been tested.

At the metabolic level, glycine—the most abundant amino acid in collagen peptides, constituting approximately 33% of the primary sequence—is one of the three constituent amino acids of glutathione (γ-glutamyl-cysteinyl-glycine); under conditions of high oxidative demand, its availability can become co-limiting for hepatic glutathione (GSH) synthesis. In a rat model of sucrose-induced insulin resistance, glycine supplementation restored glutamate-cysteine ligase (GCL/γ-GCS) expression, increased the GSH/GSSG ratio by 130%, reduced protein carbonyl and thiobarbituric acid reactive substance (TBARS) levels, and reversed the pathological serine phosphorylation of insulin receptor substrate-1 (IRS-1), thereby improving insulin sensitivity [33,34,41,42]. These findings establish a direct mechanistic link between the high glycine content of collagen hydrolysates and their observed antioxidant and insulin-sensitizing effects at the systemic metabolic level. The four receptor pathways and their downstream cascades are illustrated in Figure 4.

### 3.5. What Is Established and What Is Not

The four receptor systems above are not supported by evidence of equal strength, and the distinction matters for anyone reading this literature with a clinical decision in mind.

Three findings rest on robust experimental evidence. Orally administered collagen hydrolysate is absorbed, and a fraction of the dose reaches the circulation as intact Pro-Hyp and Hyp-Gly. Triple-helical collagen and larger collagen fragments engage α2β1 integrins, DDR1/DDR2, GPVI, and LAIR-1 in vitro, with the structural requirements described above. Glycine, delivered as a free amino acid, restores hepatic glutathione synthesis in rodent models of oxidative stress.

Two findings are demonstrated, but with a ligand that is not the absorbed fraction. The anabolic chondrocyte response to α2β1 engagement and the immunosuppressive LAIR-1 signal both require multivalent triple-helical presentation. Neither has been reproduced with monomeric Pro-Hyp or Hyp-Gly at concentrations attainable in plasma after oral dosing.

Three propositions remain hypothetical. That circulating di-/tripeptides engage the structure-dependent surface receptors directly is unproven. That DDR signaling is modified by dietary collagen in a living animal has not been tested. That the antioxidant and epigenetic effects attributed to commercial collagen hydrolysate are produced by the same sequences and concentrations used in the source studies is assumed rather than shown. The receptor biology in this section therefore describes how cells sense collagen, not how a supplement works.

## 4. Species Comparisons and Translational Relevance

### 4.1. Mammalian Species Comparison: Conservation of the PEPT1/PEPT2 System

The translational applicability of BCP research from human models to veterinary species rests primarily on the degree to which the mechanisms of peptide absorption and receptor interaction are conserved. As detailed in Section 2.4, the PEPT1/PEPT2 transporter system is highly conserved across humans, dogs (*Canis lupus familiaris*), pigs (*Sus scrofa*), and non-human primates, with cloned canine and porcine orthologs sharing the structural and functional features of the human transporter. This high conservation provides a strong mechanistic justification for expecting comparable systemic bioavailability and circulating peptide profiles across these species following equivalent oral doses, although formal species-specific pharmacokinetic studies in dogs and cats remain a significant gap in the literature [10].

Dogs and cats present with specific physiological differences from humans in their gastrointestinal tracts that may modulate CH bioavailability [31,32]. Cats, as obligate carnivores, have evolved digestive physiology optimized for a diet extremely high in animal protein, with documented adaptations in amino acid metabolism that may create different amino acid substrate dynamics following CH supplementation. Dedicated pharmacokinetic studies employing validated LC-MS/MS methodology are essential before direct dose extrapolation from human clinical data is attempted.

From the receptor perspective, the integrin α2β1, DDR1/DDR2, GPVI, and LAIR-1 receptor families are all phylogenetically ancient and highly conserved across mammalian species. The structural collagen-binding domains of these receptors are maintained across human, canine, equine, and feline proteomes. Sequence conservation of the ligand-binding domains establishes that these receptors are present in the target species. It does not establish equivalent ligand affinity, receptor density, tissue distribution, or downstream response, and no study has compared receptor-level responses to collagen fragments across canine, equine, feline, and human cells.

The knowledge gaps are not confined to dogs and cats. In horses, collagen hydrolysate is in established clinical use, yet no pharmacokinetic study of oral administration has been published in the species. In pigs and poultry, the peptide transporter biology is characterized, but the absorption of collagen-derived di- and tripeptides has not been measured under production conditions. In cats, no data exist at any level. The pharmacokinetic priority stated below therefore extends across the veterinary species in which these products are already sold.

### 4.2. Marine Collagens: Special Biochemical Properties and Veterinary Applications

Marine collagen represents a biochemically distinct sub-class of collagen raw materials with a specific property profile that is increasingly exploited in veterinary nutraceutical development. The most commercially significant marine sources include fish skin and scales (predominantly from salmonids, tilapia, and various gadiformes), jellyfish (*Rhopilema* spp.), shark cartilage, and sea cucumber [20].

The primary biochemical distinction of marine collagens is their lower thermostability, a consequence of the relatively lower content of Pro and Hyp residues in their triple-helical domains. This property is advantageous in oral supplementation contexts because it results in easier enzymatic hydrolysis, higher degrees of hydrolysis at equivalent enzyme concentrations, and potentially superior initial peptide solubility. Marine collagens also carry a substantially lower risk of mammalian pathogen transmission—including BSE, FMD, and TSEs—compared to bovine or porcine sources, and are considered free of animal pathogens [20,22].

Purified marine peptide fractions show several activities in vitro that exceed those of mammalian-derived preparations assayed under the same conditions: (1) superior antioxidant activity across multiple mechanistic assays including DPPH radical scavenging, superoxide anion scavenging, hydroxyl radical scavenging, and metal ion chelation; (2) documented melanogenesis-inhibitory activity through suppression of intracellular tyrosinase expression and activity; and (3) demonstrated ACE-inhibitory activity from jellyfish and eel-derived peptide sequences [20,21]. These activities were measured on chromatographically purified fractions and on synthetic sequences, not on commercial marine collagen supplements, whose peptide composition differs and is not routinely characterized.

### 4.3. Methodological Considerations: Limitations and Confounders

A comprehensive and critical evaluation of the BCP field requires explicit acknowledgment of the methodological limitations that constrain direct extrapolation of experimental findings to clinical applications.

#### 4.3.1. In Vitro Artefacts and Supraphysiological Concentrations

A substantial proportion of BCP receptor-binding and cell-signaling studies have been conducted at peptide concentrations (typically 0.1–1 mg/mL) that substantially exceed the circulating Pro-Hyp concentrations observed in human pharmacokinetic studies after oral ingestion of standard doses (typically 1–10 μg/mL at Cmax). Caution must therefore be exercised in assuming that in vitro mechanistic findings directly predict the magnitude of in vivo effects at physiologically achievable concentrations [18]. A second structural limitation compounds the concentration problem: the canonical collagen receptors (integrins, DDRs, GPVI, LAIR-1) require the triple-helical conformation or multivalent Gly-Pro-Hyp presentation for high-affinity binding, whereas the orally bioavailable fraction consists of monomeric di- and tripeptides. Direct receptor engagement by these small peptides at achievable plasma concentrations therefore remains unproven, and several of the receptor-level effects described above are most securely demonstrated for matrix-presented or fragment-level collagen rather than for circulating Pro-Hyp or Hyp-Gly.

#### 4.3.2. Endotoxin Contamination

Bacterial lipopolysaccharide (LPS) contamination of peptide preparations used in cell culture experiments can independently induce innate immune activation through TLR4/NF-κB signaling and confound immunomodulatory outcome measures—an effect demonstrated even at LPS concentrations below 10 pg/mL [43,44]. Rigorous endotoxin testing using the *Limulus* amebocyte lysate (LAL) assay is an essential quality control step that is not uniformly reported in the published BCP literature.

#### 4.3.3. Product Heterogeneity

Commercial CH products are complex and heterogeneous mixtures of peptides and free amino acids. Differences in raw material source, extraction method, enzyme selection, degree of hydrolysis, and post-processing treatments result in products with vastly different peptide molecular weight distributions and bioactive peptide profiles, even among products marketed under the same generic label. This heterogeneity makes direct cross-study comparisons extremely challenging [4,10].

#### 4.3.4. Dose Definition Challenges

The optimal therapeutic dose of CH is not standardized and varies considerably across studies and species. Most veterinary clinical trials have used fixed-dose protocols rather than weight-adjusted dosing regimens, potentially introducing systematic error in dose-response assessments, particularly in studies enrolling dogs across a wide bodyweight range [10].

#### 4.3.5. Allergenicity and Hypersensitivity

Although collagen hydrolysates are generally considered to have a low allergenic potential due to the disruption of conformational epitopes during enzymatic hydrolysis, dogs with established protein-responsive or food-reactive enteropathies may exhibit sensitivity to collagen hydrolysates derived from specific species. In such cases, a marine- or poultry-derived collagen source may represent a safer alternative, though systematic data on the relative allergenicity of collagen hydrolysates in companion animals are currently lacking [10].

#### 4.3.6. Publication Bias

Positive findings dominate the collagen supplementation literature. A substantial share of both preclinical and clinical studies is funded, supplied, or co-authored by manufacturers—the pivotal canine trial cited throughout this series was funded by a collagen manufacturer, and one of its authors is a co-inventor on collagen peptide patents—trial registration is inconsistent, and null results are seldom published. No formal assessment of publication bias in this field has been reported, so the size of the distortion cannot be estimated. The effect sizes summarized in this review should be read as upper bounds rather than as central estimates.

## 5. Conclusions

Bioactive collagen peptides constitute a molecularly defined class of nutraceutical agents whose physiological relevance extends beyond the simple provision of structural amino acid substrates. The pharmacokinetic, receptor, and veterinary clinical data reviewed here support a plausible mechanistic model rather than an established causal pathway. Several of its steps remain untested, in particular the link between the absorbed di-/tripeptide fraction and the structure-dependent collagen receptors.

Orally administered collagen hydrolysate is efficiently absorbed, and a fraction of the dose reaches the circulation as intact bioactive di- and tripeptides—predominantly Pro-Hyp and Hyp-Gly—through the highly conserved PEPT1/PEPT2 transporter system, the remainder as free amino acids [18,19]. Collagen and its fragments are sensed by at least four receptor classes—α2β1 integrins driving anabolic ECM signaling [35], DDR1/DDR2 receptors modulating matrix homeostasis and cellular metabolism [1], and the hematologic and immune receptors GPVI and LAIR-1/LAIR-2 [36]—each dependent on triple-helical or repetitive-motif presentation, together with the intracellular Keap1–Nrf2 antioxidant pathway [38]. A central open question is how far the orally absorbed di-/tripeptide fraction engages these structure-dependent receptors directly, as opposed to acting through intracellular and substrate-level mechanisms.

The high conservation of the PEPT1/PEPT2 system across humans, dogs, and pigs provides strong mechanistic support for the translational applicability of human pharmacokinetic data to companion animal medicine. Dedicated species-specific bioavailability and pharmacokinetic studies, employing validated LC-MS/MS methodology for canine biological matrices [45,46,47,48], should be considered a high research priority. The adoption of standardized outcome measures across future BCP clinical trials—including validated force plate kinetic analysis, validated owner-assessed pain questionnaires such as the CBPI, and standardized serum cartilage biomarker panels—would enable rigorous cross-study meta-analyses and substantially strengthen the evidence base [14].

Standardized outcome measures alone will not make trials comparable. Future studies should also report a standardized characterization of the preparation used: raw material and species of origin, extraction and hydrolysis conditions, enzyme and degree of hydrolysis, peptide molecular-weight distribution, quantified Pro-Hyp and Hyp-Gly content, and endotoxin concentration measured by a validated assay. Without these data, a null result cannot be distinguished from an inactive batch, and meta-analysis across trials is not defensible.

Part II of this series turns from mechanism to application. It addresses the gut–collagen peptide axis and intestinal barrier function, microbial bile acid remodeling and FXR/TGR5 signaling, the enteroendocrine (GLP-1, PYY) response, musculoskeletal applications in dogs, horses, cats, and livestock, taurine-responsive dilated cardiomyopathy as a mechanistically separate input to the same bile acid pool, and the hepatic, oncological, and neurological literature, with the same stratification of evidence applied throughout.

## Figures and Tables

**Table 1:** Comparison of the three collagen-based product classes discussed in this review. Doses are those used in the cited studies and are not dose recommendations. Abbreviations: CH, collagen hydrolysate; BCP, bioactive collagen peptide; UC-II, undenatured type II collagen; GALT, gut-associated lymphoid tissue; MW, molecular weight; Da, Dalton; kDa, kilodalton; OA, osteoarthritis; Treg, regulatory T cell; ECM, extracellular matrix; RCT, randomized controlled trial.

Property	Collagen Hydrolysate (CH)	Bioactive Collagen Peptides (BCPs)	Undenatured Type II Collagen (UC-II)
**Definition**	Enzymatically hydrolyzed, fully soluble collagen: a mixture of oligopeptides and free amino acids	The subset of sequences within or released from CH for which biological activity has been measured	Non-hydrolyzed type II collagen retaining its native conformation
**Structure**	Denatured; no triple helix	Di- and tripeptides, principally Pro-Hyp and Hyp-Gly	Intact triple helix; conformational epitopes preserved
**Typical MW**	2–6 kDa (mean); mostly <3000 Da	200–350 Da	Intact protein
**Site of action**	Intestinal lumen and, after absorption, systemically	Systemic: cartilage, dermal ECM, endothelium	Intestinal mucosa (Peyer’s patches)
**Principal mechanism**	Substrate provision plus release of BCPs during digestion	Intracellular routes (Keap1–Nrf2, HDAC/HAT) and glycine-dependent glutathione synthesis; direct receptor engagement unresolved	Oral tolerance induction via GALT; Treg-mediated suppression of cartilage-directed autoimmunity
**Absorption**	Partly as free amino acids, partly as di-/tripeptides via PEPT1/PEPT2	Via PEPT1/PEPT2; plasma peak at 60–120 min	Not systemically absorbed; acts locally
**Dose in cited canine trials**	240 ± 95 mg/kg BW per day [14]	Not dosed separately; delivered within CH	10 mg per dog per day [11,15]
**Veterinary evidence**	Canine and equine OA: controlled trials with objective kinetics	Mechanistic only; no BCP-specific veterinary trial	Canine and equine OA: controlled trials
**Principal limitation**	Product heterogeneity; no lot-level characterization	Plasma concentrations far below in vitro effective concentrations	Mechanism inferred from oral tolerance models; no head-to-head trial against CH

**Table 2:** Comparison of Key Industrial Processing Parameters and Resulting Bioactive Peptide Profiles Across Major Collagen Hydrolysate Sources. All values represent approximate ranges reported in the literature; exact values are highly dependent on proprietary processing protocols. The poultry/Type II column refers to undenatured type II collagen (UC-II), which is not a hydrolysate; its molecular-weight value reflects the intact triple-helical protein and is included for comparison only. Abbreviations: MW, molecular weight; Pro-Hyp, prolyl-hydroxyproline; ACE, angiotensin-converting enzyme; BSE, bovine spongiform encephalopathy; kDa, kilodalton. Values are approximate literature ranges, not product specifications, and should not be used for formulation or dose calculation.

Parameter	Bovine (Hide)	Porcine (Skin)	Marine (Fish Skin)	Poultry (Type II, Sternum)
**Primary collagen type**	I, III	I, III	I	II
**Typical extraction method**	Acid/alkaline + hydrothermal	Acid + enzymatic	Low-temp enzymatic	Pepsin
**Optimal hydrolysis enzyme**	Alcalase/Protamex	Trypsin/Pepsin	Trypsin (3 h optimal)	Pepsin
**Mean MW output (kDa)**	2–5	2–5	1–3	Not a hydrolysate; intact triple-helical protein
**Denaturation temp (°C)**	~36	~35	~16	~34
**Pro + Hyp content (% AA)**	~22	~22	~17–18	~21
**Circulating Pro-Hyp (ΔCmax)**	~2.7 μg/mL	~3.8 μg/mL	~2.2 μg/mL	n/a
**Primary bioactivities**	ECM anabolic, antithrombotic	ECM anabolic	Antioxidant, ACE-inhibitory	Oral tolerization (GALT)
**BSE/zoonotic risk**	Yes (low with processing)	Low	None for BSE; low environmental risk	Low
**Mechanism**	Receptor-mediated (integrin/DDR2)	Receptor-mediated	Nrf2/Keap1, GPVI	Immune tolerance (Tregs)
**Key references**	Virgilio et al., 2024; Osawa et al., 2018; [18,19]	Virgilio et al., 2024; Dobenecker et al., 2024 [14,18]	Coppola et al., 2020; Zhuang et al., 2009 [20,21]	Deparle et al., 2005; Gupta et al., 2009 [11,12]

## Data Availability

No new data were created or analyzed in this study. Data sharing is not applicable to this article.

## Abbreviations

The following abbreviations are used in this manuscript:

ACE	angiotensin-converting enzyme
ARE	antioxidant response element
AUC	area under the curve
BA	bile acid
BCP	bioactive collagen peptide
BSE	bovine spongiform encephalopathy
CAT	catalase
CH	collagen hydrolysate
DPPH	2,2-diphenyl-1-picrylhydrazyl
DDR1/2	discoidin domain receptor 1/2
ECM	extracellular matrix
FMD	foot-and-mouth disease
FXR	farnesoid X receptor
GALT	gut-associated lymphoid tissue
γ-GCS	γ-glutamylcysteine synthetase
GPH	glycine-proline-hydroxyproline
GPVI	glycoprotein VI
GR	glucocorticoid receptor
GSH	glutathione
GSSG	oxidized glutathione
HAT	histone acetyltransferase
HDAC	histone deacetylase
HMW	high molecular weight
HO-1	heme oxygenase-1
Hyp	hydroxyproline
Hyp-Gly	hydroxyprolyl-glycine
IRS-1	insulin receptor substrate-1
ITIM	immunoreceptor tyrosine-based inhibitory motif
Keap1	Kelch-like ECH-associated protein 1
kDa	kilodalton
LAL	*Limulus* amebocyte lysate
LAIR-1/2	leukocyte-associated immunoglobulin-like receptor 1/2
LC-MS/MS	liquid chromatography-tandem mass spectrometry
LMW	low molecular weight
LPS	lipopolysaccharide
MAPK	mitogen-activated protein kinase
MMP	matrix metalloproteinase
NF-κB	nuclear factor kappa-light-chain-enhancer of activated B cells
Nrf2	nuclear factor erythroid 2-related factor 2
PEPT1/2	peptide transporter 1/2 (SLC15A1/A2)
Pro	proline
Pro-Hyp	prolyl-hydroxyproline
SASP	senescence-associated secretory phenotype
SHP-1/2	Src homology 2-containing protein tyrosine phosphatase 1/2
SOD	superoxide dismutase
TBARS	thiobarbituric acid reactive substances
TGF-β	transforming growth factor-beta
TGR5	Takeda G-protein-coupled receptor 5
TIMP	tissue inhibitor of metalloproteinases
TLR4	Toll-like receptor 4
TSE	transmissible spongiform encephalopathy
UC-II	undenatured type II collagen
